# Effect of new biological patch in repairing intrauterine adhesion and improving clinical pregnancy outcome in infertile women: study protocol for a randomized controlled trial

**DOI:** 10.1186/s13063-022-06428-0

**Published:** 2022-06-18

**Authors:** Wen-Juan Pang, Qing Zhang, Hai-Xia Ding, Ning-Xia Sun, Wen Li

**Affiliations:** 1 Reproductive Medicine Center, Second Affiliated Hospital of Naval Medical University, No. 415 of Fengyang Road, Huangpu District, Shanghai, 200003 China; 2 International Peace Maternity and Child Health Hospital, School of Medicine, Shanghai Jiao Tong University, No. 910 of Hengshan Road, Xuhui District, Shanghai, 200030 China

**Keywords:** Intrauterine adhesion, Endometrial fibrosis, Extracellular matrix, In vitro fertilization–embryo transfer

## Abstract

**Background:**

Endometrial fibrosis caused by intrauterine adhesion (IUA) can lead to hypomenorrhea, amenorrhea, and even infertility and abortion. The postoperative recurrence rate of severe IUA remains high, giving rise to low pregnancy rates. An extracellular matrix (ECM) scaffold, a new biological material that can promote cell proliferation and differentiation at lesions, has been widely used in general surgery and neurosurgery. The present study applied ECM scaffolds in obstetrics and gynecology for the first time to improve endometrial fibrosis, repair severe IUA, and improve pregnancy outcomes for infertile patients.

**Methods:**

This paper presents a prospective randomized single-blind controlled superiority study of infertile women aged ≤40 years with IUA. According to the scoring criteria for IUA established by the American Fertility Society, patients with moderate or severe IUA were randomized into two groups at a ratio of 1:1; patients in the experimental group were treated with an ECM scaffold (small intestinal submucosa [SIS]) + intrauterine balloon, while patients in the control group were treated with an intrauterine balloon only. A hysteroscopic examination of adhesion repair was performed again after 2 months of postoperative hormone replacement therapy. Endometrial tissue was sampled during the two operations, and immunohistochemistry was used to observe endometrial and microvascular proliferation. After thawing and resuscitation, a postoperative frozen embryo transfer was performed on the participants in both groups, and their endometrial thickness, intrauterine volume, endometrial vascularization flow index, endometrial flow index, and uterine artery blood flow resistance were evaluated by 3D ultrasonography. The rates of embryo implantation, clinical pregnancy, and early spontaneous abortion were observed.

**Discussion:**

The ECM scaffold (SIS) + intrauterine balloon method was able to repair endometrial fibrosis and improve IUA. This new technique represents a novel treatment method for improving the pregnancy outcome of infertile patients with moderate/severe IUA.

**Trial registration:**

Chinese Clinical Trial Register ChiCTR2100052027. Registered on October 14, 2021.

## Background

Infertility affects 48.5 million couples worldwide with a prevalence estimated at 3.5–16.7% in low- and middle-income countries and up to 30–40% in sub-Saharan Africa [1]. Intrauterine adhesion (IUA) accounts for 8% of the causes of infertility and is also a common cause of secondary infertility [2]. Two-thirds of female infertility cases are caused by repeated embryo implantation failure (RIF) during in vitro fertilization–embryo transfer as a result of endometrial factors, indicating the serious impact of an endometrial injury on female reproductive function [3].

In addition to infertility, an endometrial basal layer injury can also lead to partial or total intrauterine occlusion, resulting in amenorrhea, hypomenorrhea, periodic abdominalgia, recurrent abortion, placental abnormalities, and other symptoms. Accordingly, endometrial injury has become a major reproductive system disease that seriously threatens female reproductive health.

Endometrial injury can easily cause IUA, which primarily manifests as an endometrial basal layer injury caused by trauma, infection, and other factors, resulting in the dysfunction of functional endometrial repair; in turn, this will lead to the proliferation of fibrous connective tissue and endometrial fibrosis as well as partial or total intrauterine occlusion [2, 4]. According to the consensus of Chinese experts on the clinical diagnosis and treatment of intrauterine adhesions [5], at present, for patients suffering from infertility, recurrent abortion, and oligomenorrhea and for those with special fertility requirements, transcervical resection of adhesion (TCRA) can be conducted as a preferred treatment (recommendation level: C). Moreover, TCRA combined with an intrauterine device (IUD), Foley balloon support, or estrogen and progesterone replacement therapy has shown degrees of anti-adhesion value, although their effects are variable. An IUD placement after moderate and severe IUA could reduce the formation rate of re-adhesion to 35% [6] (evidence level: I). But meta-analysis showed that the menstrual improvement rate ranged between 28.5 and 100%. Several studies have indicated significant differences in this regard [7]. In addition, although an intrauterine balloon can make the total effective rate of IUA repair reach 71.67% [8] and improve the menstrual rate up to 81.4–95% [9, 10], the shape of the balloon used in clinics is not suitable for the intrauterine cavity, which makes it difficult to block the wound completely. Moreover, if the intrauterine balloon is not placed properly, intimal ischemia and necrosis may occur, which will impact intimal regeneration and repair. In particular, for patients with severe IUA, due to endometrial basal layer damage, reduced residual normal endometrial tissue, and poor regeneration ability, the recurrence rate of postoperative adhesion can still be as high as 62.5%, while the success rate of pregnancy will range between only 22.5 and 33.3% [11]. Therefore, establishing an anti-adhesion method that can promote the repair of endometrial injury is of great significance.

Advances in the field of tissue engineering present new opportunities in the development of regenerative medicine and provide new directions for the treatment of endometrial injuries. As a new medical material, the primary structure of a biological patch comprises an extracellular matrix (ECM) fibrous scaffold made up of collagen, elastic fiber, glycoprotein, laminin, and proteoglycan; together, these proteins provide an ideal scaffold for the repair of defective tissue. In recent years, the effect and feasibility of using biological patches to strengthen and repair fascia tissue in pelvic floor reconstruction have been confirmed [12]. The present authors’ research applied an ECM scaffold to repair endometrial injuries in a rat model and increase the pregnancy rate among rats [13]. The results were consistent with the effect of small intestinal submucosa (SIS), as reported by Ho, in the repair of vaginal injury in a rat model [14].

This paper proposes a new scheme for the prevention of IUA and endometrial repair. An ECM scaffold (i.e., SIS, provided by Cook Inc., USA) was placed, based on TCRA, and combined with an intrauterine balloon to explore the effect of the ECM scaffold on endometrial cell proliferation and anti-fibrosis and analyze its efficiency in improving the clinical pregnancy outcomes of patients with moderate and severe IUA infertility.

### Experimental design

**Fig. 1:**
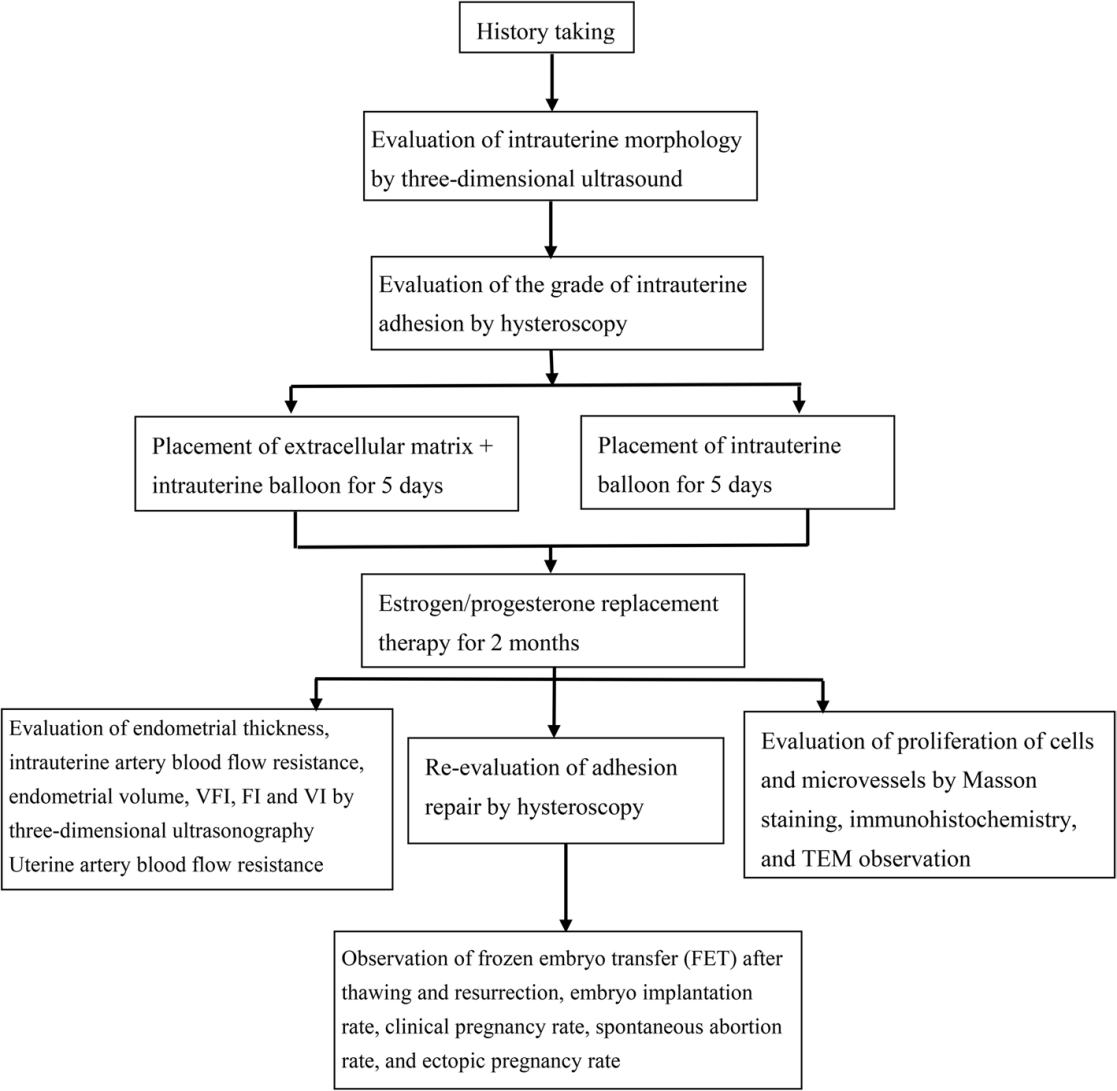
Study design flowchart

The current paper presents a prospective randomized single-blind controlled superiority study that evaluated the ability of ECM scaffolds in the repair of endometrial fibrosis. Patients with infertility with a history of secondary hypomenorrhea, a thin endometrium, and RIF (excluding factors such as embryo quality) who suffered from IUA were recruited from the Reproductive Medicine Center of Shanghai Changzheng Hospital, China. According to the scoring criteria for IUAs established by the American Fertility Society (AFS) shown in Table 1 [15], patients with moderate or severe IUAs were randomized into the ECM scaffold + intrauterine balloon implantation group or the intrauterine balloon implantation group at a ratio of 1:1. The study design process is shown in Fig. 1.

**Table 1:** AFS scoring criteria for intrauterine adhesion (1988)

Category	Score
Extent of intrauterine adhesion	
<1/3	1
1/3–2/3	2
>2/3	4
Type of adhesion	
Filmy	1
Filmy and dense	2
Dense	4
Menstrual pattern	
Normal	0
Hypomenorrhea	2
Amenorrhea	4

Total AFS score = score for extent of cavity involved + score for type of adhesion + score for menstrual pattern

severe: 9–12 points; moderate: 5–8 points; mild: 1–4 points

*AFS* American Fertility Society

### Evaluation criteria

The evaluation criteria for inclusion in the protocol were as follows.

1. Secondary hypomenorrhea: The menstrual blood volume was less than 20 ml or had decreased by 1/3 of the original menstrual blood volume, and the menstrual cycle length was less than 3 days if the previous menstrual bleeding duration and volume had been normal. Patients could calculate this according to the number of sanitary napkins they had used.
2. Diagnosis of infertility: Patients who failed to become pregnant successfully with a normal sex life without taking contraceptive measures for more than 1 year.
3. A “thin endometrium” meant that the thickness of the endometrium was less than the threshold thickness for becoming pregnant. Currently, there is no unified standard for this threshold. Most scholars posit that an endometrial thickness of <7 mm as measured by transvaginal ultrasound on the day of human chorionic gonadotropin (hCG) could be considered a thin endometrium [16].
4. An IUA diagnosis under 3D ultrasound [17] in which type I indicates a clear endometrium and a discontinuous endometrial line, type II denotes a mild intrauterine separation with a separation diameter less than 1 cm, type III refers to an unclear endometrium with a thickness of less than 0.2 cm that is not demarcated from the surrounding muscle layer, and type IV involves severe intrauterine separation with a separation diameter greater than 1 cm. A diagnosis could be made if one of the above characteristics was present.
5. “Repeated embryo implantation failure” refers to failure to achieve a pregnancy following 2–6 IVF cycles, in which more than 10 high-grade embryos were transferred into the uterus [18].

The protocol complies with the principles of the Declaration of Helsinki as well as Good Clinical Practice (GCP) guidelines. The hospital’s ethics committee approved the study protocol. The trial was registered in the Chinese Clinical Trial Registry.

The inclusion criteria were as follows:

1. Patients aged ≤ 40 years
2. Patients with a body mass index = 18–27.9 kg/m^2^
3. Patients with secondary decreased menstruation
4. Patients with a thin endometrium
5. Patients with RIF
6. Patients who met the diagnostic criteria of the World Health Organization for infertility (normal regular sex life, no contraception, no pregnancy for 1 year)
7. Patients who had undergone in vitro fertilization (IVF)/intracytoplasmic sperm injection (ICSI) treatment cycle and obtained at least two high-quality embryos (including fallopian tube factors, ovulation disorders, male severe oligozoospermia, severe asthenospermia, and severe teratospermia)
8. Patients with a clinical diagnosis of moderate/severe IUA (conforming to the AFS 1988 classification criteria) [15]
9. Patients who agreed to undergo hysteroscopy and postoperative re-examination
10. Patients who were able to understand and cooperate in the clinical study process and comply with the requirements of the study protocols until its completion
11. Patients who agreed to be included in the clinical study and who voluntarily provided written informed consent accordingly

The exclusion criteria were as follows:

1. Patients with irregular vaginal bleeding of an unknown cause
2. Patients with ovarian, pituitary, or hypothalamic amenorrhea
3. Patients with abnormal uterine development
4. Patients with uterine fibroids of >2 cm, adenomyosis, or severe pelvic endometriosis
5. Patients with a history of malignant or borderline tumors
6. Patients with breast hyperplasia or other related diseases
7. Patients with a history of pelvic tuberculosis or acute infection of the genitourinary system
8. Patients with serious cardiovascular, cerebrovascular, respiratory, or digestive diseases
9. Patients with a history or family history of severe thrombotic diseases
10. Patients with severe liver or kidney dysfunction or immune system disorders
11. Patients with psychosis or a serious mental illness
12. Patients with other diseases that were incompatible with human-assisted reproductive technology
13. Patients with a history of allergic reactions to porcine-derived products
14. Patients who could not accept porcine-derived materials due to religious beliefs or ethnicity
15. Other patients who were considered ineligible for the study

The withdrawal criteria were as follows:

1. The participant or their legal guardian voluntarily requested withdrawal
2. Violation of the inclusion/exclusion criteria
3. Withdrawal in the case of other unexplained serious complications
4. Withdrawal from the study in the case of becoming pregnant during the treatment
5. The sponsor of the study proposed discontinuing the study for safety reasons
6. Subjects whose participation in this study was discontinued by the hospital’s ethics committee
7. Subjects whom investigators considered unsuitable for participation in the study

### Screening of infertility patients with intrauterine adhesions

Patients with infertility with secondary hypomenorrhea were evaluated by 3D ultrasound for endometrial thickness and morphology on days 3–7 after menstruation, and the intrauterine volume, endometrial vascularization flow index (VFI), endometrial flow index (FI), and endometrial vascularization index (VI) were recorded. According to the patients’ medical history and 3D ultrasound imaging examinations, infertile women aged ≤ 40 years who suffered from IUAs were screened and admitted to the hospital for adhesiolysis. Following admission, the patients were collected for a review of their general circumstances and medical history, which included blood pressure, heart rate, pulse and respiration, menstruation, menstrual and obstetrical histories, past medical history, operation history, and family medical history.

### Study protocols

#### Hysteroscopic evaluation

Following perfect preoperative preparation and the provision of signed consent, the hysteroscopy-guided separation of IUAs was performed under general anesthesia 3–7 days after the end of menstruation. During the surgery, the bladder lithotomy position of the patient was taken. A hysteroscopy operating mirror (Storz, Germany) (dimensions: 5.5–7.5 mm) equipped with hysteroscopic direct-vision micro scissors was used; the normal saline perfusion system continued to expand the uterus at a pressure of 120–140 mmHg, and the cervix was expanded by 5.5–7.5 mm. The hysteroscopy was performed with IUA having been scored before surgery. Cooled knife-sharp separation was used in all of the surgeries, with difficult procedures being performed under the guidance of B-scan ultrasonography. The success criteria were to remove scar tissue, preserve normal intima, normal or basically normal morphology of uterine cavity, and clear display of bilateral fallopian tube openings. For severe intrauterine adhesion, B-ultrasound should be used to monitor the thickness of uterine myometrium during operation to prevent uterine perforation.

Based on the AFS scoring criteria for IUA [15] (Table 1), patients with moderate/severe IUA were randomized into the experimental group (which included the placement of an ECM scaffold [SIS] and a Foley balloon) or the control group (which included the placement of a Foley balloon) at a ratio of 1:1. In the control group, the Foley balloon was placed after surgery, and normal saline was injected into the balloon according to the size of the uterine cavity. When obvious resistance occurred, the saline injection was stopped (normal saline was 3~5 ml); a drainage bag was connected, and the balloon was removed after 5 days (the recommended prevention time of the balloon by experts in China was 5~7 days [5], recommendation level C). To reduce the infection rate among the patients, a 5-day balloon retention time was implemented in combination with the actual situation of our hospital. Antibiotics were used to prevent any infections from occurring after surgery. In the experimental group, the ECM patch was cut into a 2 × 5-cm rectangle. Fold the patch in half along the midpoint line of the short side, then open it, and place the unfilled balloon on the fold line. Cover the other half of the patch on the balloon, wrap it tightly, clamp it with long curved forceps, and place it into the uterine cavity. Other procedures were the same as those in the control group.

#### Postoperative hormone replacement therapy

Patients in the experimental and control groups all received anti-infection treatment following surgery. Additionally, 2-mg exogenous estrogen (twice daily) and 50-mg aspirin (once daily) were given for 14 consecutive days (starting on the first day after surgery), and 10-mg dydrogesterone (twice daily) was added on day 10 after commencing estrogen therapy. Postoperative hormone replacement therapy (HRT) was provided in two cycles.

#### Hysteroscopic re-examination

Patients in both the experimental and control groups received a hysteroscopic re-examination 3–7 days after the end of the second menstruation to evaluate the IUA repair. Postoperative HRT was repeated (the same as stated above). According to the evaluation standard for uterine cavity repair, if the second hysteroscopy showed that uterine cavity adhesion had not been recovered (i.e., there was no obvious change between the uterine cavity’s shape and that before separation, based on hysteroscopy), or the adhesion was more serious, full adhesiolysis was performed again, and additional anti-adhesion methods, such as inserting an IUD and replacing estrogen and progesterone for one month, were implemented.

#### Tissue repair evaluation

An endometrial biopsy was performed following IUA lysis. A small amount of endometrial tissue was taken for pathological and endometrial repair evaluations. The degree of endometrial fibrosis and the number of proliferating cells and microvessels in the two groups were observed using Masson’s staining and immunohistochemistry techniques to evaluate the effect of the ECM scaffolds on the proliferation of endometrial cells. A transmission electron microscope was used to observe the ultrastructure of the endometrial cells.

##### Sampling of endometrial tissue

The endometrial samples of infertile patients with moderate/severe IUA were obtained under hysteroscopy. Some of the samples were immersed in 4% paraformaldehyde and fixed for 24 h, while others were frozen rapidly in liquid nitrogen and stored at −80°C for later use.

##### Paraffin embedding and section preparation of sample tissues

These procedures included the following: (1) tissue gradient dehydration, (2) tissue clarification, (3) wax infiltration, (4) embedding, and (5) sectioning. The sections were floated in warm (45°C) water in a spreading machine, picked up with slides once the tissue had been fully flattened, baked at 60°C, removed 2 h later, and stored at a normal temperature (25°C) for later use.

##### Masson’s staining

This procedure involved the following steps: (1) dewaxing and hydration, (2) hematoxylin nuclear staining, (3) Ponceau staining, (4) treatment with phosphomolybdic acid, (5) aniline blue staining, (6) differentiation, (7) dehydration and clarification, (8) mounting, and (9) optical microscopy, image collection, and analysis.

##### Determination of endometrial cell proliferation

To determine whether the ECM could promote endometrial cell proliferation, Ki67 immunohistochemical staining was conducted. Von Willebrand factor immunohistochemical staining in the endometrial injury region was conducted to observe the regeneration and distribution of microvessels in the endometrium. At a magnification of ×40, the proliferative cell count of three fields was randomly selected and averaged.

#### Frozen embryo transfer after thawing

Patients were given 4 mg of exogenous estrogen on the 2nd/3rd day of menstruation at a maximum dose of 8 mg for a total of 15–20 days. When the endometrial thickness was ≥7 mm, progesterone was given for endometrial transformation, and embryo transfer was generally performed on days 20–22. Three-dimensional ultrasonography was performed to evaluate the endometrial thickness and morphology, intrauterine volume, endometrial VFI, FI, VI, and uterine artery blood flow resistance. Between 1 and 2 high-quality embryos were transplanted. Blood beta (β)-hCG and progesterone were measured 14 days after embryo transplantation. At 35 days after embryo transfer, B-scan ultrasonography was performed to check the clinical pregnancy status.

#### Embryo scoring criteria

1. The criteria for embryo evaluation at the cleavage stage and embryo scoring were as follows:
  Grade I: Blastomeres that were uniform in size and free of fragments
  Grade II: Blastomeres that were uniform or slightly uneven in size with fragments < 20%
  Grade III: Blastomeres that were uniform or slightly uneven in size with fragments <20–50%
  Grade IV: Blastomeres that were uneven in size with fragments >50%

Grades I and II indicated high-quality embryos, and grades I, II, and III indicated transplantable embryos.

2) Blastula evaluation criteria. Blastocysts could be classified as grades I–VI according to the degree of expansion and hatching, as follows:
  Grade I early blastula: The volume of the blastular cavity was smaller than half of the total volume of the blastocyst
  Grade II blastula: The volume of the blastular cavity was larger than half of the total volume of the blastocyst
  Grade III completely expanded blastula: The blastular cavity occupied the entire lumen
  Grade IV blastula after expansion: The volume of the blastular cavity was significantly larger than the early blastula, and the zona pellucida was thinner
  Grade V incubating blastula: The blastula incubated from the rupture of the zona pellucida
  Grade VI hatched blastula: the blastulae emerged completely from the zona pellucida.
  Grades III–VI blastulae were scored for inner cell mass (ICM) and trophoblast ectoderm cells.

The ICM scores were as follows:

- Grade A: A large number of cells, binding tightly
- Grade B: Fewer cells, binding loosely
- Grade C: Very few cells

The trophoblast ectodermal cell scores were as follows:

- Grade A: A large number of cells distributed around the blastocysts
- Grade B: Fewer cells and relatively loose epithelial cells
- Grade C: Very few cells

Blastocysts with a D5 score of ≥3AA, 3AB, 3BA, or 3BB or a D6–7 score of ≥4AA, 4AB, 4BA, or 4BB were identified as high-quality blastocysts.

### Randomization and allocation

Eligible participants will be randomly assigned to the experimental group or the control group in a 1:1 ratio by central randomization performed by an independent statistician from our team. Random numbers are generated by Excel, which will generate a random number table, and the allocation sequence was registered on the Shanghai Shenkang Development Center (https://crip-ec.shdc.org.cn/html/admin/auth/login). According to the random number table, the statistician puts each grouping scheme into an opaque envelope, writes the code on the outside of the envelope, seals it, and gives it to the researcher. Once the hysteroscopic process entered the uterine cavity, the degree of uterine cavity adhesion was evaluated according to AFS. If the uterine cavity was assessed as reflecting moderate or severe uterine cavity adhesion, the itinerant nurse opened the envelope with the corresponding number. Then, the operator intervened according to the grouping scheme in the envelope. The process of unpacking the envelope was time consuming but did not affect anesthesia or hysteroscopy. The researchers were made aware of the distribution following the hysteroscopic surgery.

### Outcome measures

#### Primary outcome measures

The response rate of the repair of moderate/severe IUAs served as the main evaluation index.

The definition of an “effective repair of IUA” was considered as the total AFS of adhesion having been decreased by ≥4 points.

##### Complete response

The morphology of the endometrial cavity was returned to normal, and there was complete endometrial coverage.

##### Moderate response

The endometrial cavity reflected a normal morphology, the endometrial volume was enlarged compared with before surgery, and the IUA score was decreased by 1–2 grades, although a small amount of adhesion remained.

##### No response

No significant changes were observed in the endometrial cavity under hysteroscopy compared with before separation.

The response rate % = the number of participants with successful intrauterine repair in a group / the number of subjects in that group × 100%.

### Secondary outcome measures

These included the endometrial thickness, menstrual volume, and clinical pregnancy rate.

1. “Endometrial thickness” referred to the anterior and posterior diameter of the endometrium in the longitudinal section of the uterus, which changed with the menstrual cycle. The measurement of endometrial thickness was based on the following: In the median sagittal section of the uterine body, perpendicular to the midline of the endometrium, the maximum thickness diameter was measured between the lateral edges of the double-layer endometrium. If there was uterine effusion, the thickness and diameter of the two monolayers were measured and added to the overall total.
2. The evaluation of menstrual volume is the total amount of bleeding in each menstrual cycle. The normal menstrual volume is 20–60 ml, and more than 80 ml is considered to be excessive. Typically, this is judged by the number of sanitary napkins used. Under normal circumstances, 10–20 sanitary napkins will be used in a menstrual cycle. If the level of menstruation required fewer than 10 sanitary napkins or only one was needed, the amount of menstruation had been reduced. Based on the number of sanitary napkins that were used before the hysteroscopy, if this was more than the amount before the procedure, the amount of menstruation was considered to have increased. If there was no change in the menstrual volume compared with before the procedure, the menstrual volume was considered as having shown no improvement.
3. Blood β-hCG and progesterone were measured 14 days after embryo transfer to evaluate whether a biochemical pregnancy was present. On day 35 after embryo transfer, a vaginal ultrasound showed that there was a gestational sac in the uterus with an embryo and fetal heart beat; this was determined as a clinical pregnancy.
  Ongoing pregnancy rate = the number of clinical pregnancies ≥12 weeks/the number of embryo transfer cycles × 100%
  Clinical pregnancy rate = the number of clinical pregnancies/the number of embryo transfer cycles × 100%
  Embryo implantation rate = the total number of implanted embryos/the total number of transplanted embryos × 100%
  Ectopic pregnancy rate = the number of ectopic pregnancies/the total number of clinical pregnancies × 100%
  Early spontaneous abortion rate = the number of spontaneous abortions at a gestational age of <12 weeks/the total number of clinical pregnancies × 100%

### Safety evaluation indexes

The safety evaluation indexes included postoperative intrauterine infection (fever, abnormal vaginal discharge), the duration of vaginal bleeding, lower abdominal pain (Visual Analogue Scale/Score, VAS pain score), and allergic reactions. VAS is a commonly used scoring standard for evaluating pain intensity in the clinic. This method is classified according to the impact of pain on patients’ sleep. If pain symptoms do not affect patients’ sleep, patients belong to mild pain. If the pain symptoms will affect the patient’s sleep, but the patient can still fall asleep, the patient belongs to moderate pain. If the pain symptoms make the patient unable to sleep, the patient belongs to severe pain symptoms. Patients will be asked about the degree of pain 2–3 days after hysteroscopy.

### Sample size

The sample size calculation is based on the repair rate of moderate/severe IUA. Based on the fact that the repair rate of Foley balloon placement was 47–71.67% [8, 19], we assume that the effective rate of uterine adhesion repair can reach 80%. According to the calculation method for several cases related to the superiority study, the number of samples was estimated as 83, with *α* = 0.025 (one-sided) and *β* = 0.2. A sample size of 83 prospectively enrolled participants in each randomization arm. In consideration of a dropout rate of 20%, we will enroll 100 participants in each group response.

### Adverse events

Adverse events (AEs) referred to any adverse medical events experienced by a participant during the study; it concerned their participation in the research, regardless of its relationship with research intervention. Serious AEs (SAEs) referred to events related to a participant that occurred during the study and met any of the following criteria: death, a life-threatening event, a severe or persistent disability, requiring or prolonging hospitalization, disability, congenital malformation, or any other events considered serious by the principal investigator. All AEs were evaluated and recorded in detail. All SAEs were reported to the principal investigator within 5 days, and appropriate measures were taken immediately. Any SAEs that were not intentional and that were possibly related to study interventions were reported within 24 h. In such cases, the ethics committee determined whether an AE or SAE was related to the study intervention and whether it was necessary to breach the blinding guidelines.

### Data management

All investigators, including doctors, nurses, and research associates, attended a training workshop before the trial commenced to ensure the accuracy of both outcome assessments and data collection. All investigators were provided with a protocol and standard operating procedures. All data were collected in the standard general reporting format and recorded in the electronic data acquisition system (the CRIP system of the Shanghai Shenkang Hospital Development Center). The Shanghai Shenkang Hospital Development Center and the Shanghai Changzheng Hospital appointed data inspectors to regularly check the authenticity, accuracy, and integrity of the data to ensure the quality of the data collected.

### Statistical analysis methods

The data were analyzed using the R (v.2.1.1) tool. The measurement data were statistically presented in terms of mean ± standard deviation, maximum, minimum, mean 95% confidence interval, and median. A *t*-test was used for intergroup comparisons of data conforming to normality and homogeneity of variance, while a nonparametric test (chi-square test) was used for intergroup comparisons of data that did not conform to normality. The enumeration data were statistically represented by frequency (composition ratio). A rank–sum test was used for ordinal categorical variables, and the chi-square test was used for nominal categorical variables. Two-tailed tests were used for all statistical analyses, and a *P*-value of less than or equal to 0.05 was considered statistically significant for the tested difference.

### Ethical considerations

The protocol for this clinical trial was in line with the principles of the Declaration of Helsinki and was approved by the Biomedical research ethics committee of Shanghai Changzheng Hospital (approval document no. 2020SL031). The trial was registered in the Chinese Clinical Trial Registry (ID: ChiCTR2100052027). All of the participants were fully informed of the study protocols. Signed informed consent was obtained from all the participants before they participated in the study.

## Discussion

The endometrium is essential for a successful pregnancy. Intrauterine adhesion caused by an endometrial basal layer injury can cause hypomenorrhea, amenorrhea, periodic abdominalgia, infertility, recurrent abortion, placental abnormalities, and other symptoms. Hysteroscopy-guided adhesion separation can recover the endometrial cavity to varying degrees. However, endometrial fibrosis cannot be improved in patients with severe IUAs due to the severely damaged basal layer of the endometrium, reduced residual normal endometrial tissue, and poor regeneration ability, resulting in a high postoperative recurrence rate of IUA, even if the intrauterine volume is restored. In recent years, as a new medical material, biological patches have been widely used in clinical defective tissue repair with good application prospects. An ECM scaffold can provide physical support for the normal physiological activities of tissue cells and act as a ligand polymer for cell surface receptors, thus forming a unique cellular microenvironment and regulating cellular biological functions. The ECM can regulate the homeostasis of the tissue microenvironment through synthesis, degradation, reorganization, and chemical modification; furthermore, it can act as a repository of signal molecules, including inflammatory transmitters, to transfer external stimuli to proliferating, differentiating, and migrating cells to achieve immune regulation [20]. At the early stage of body development, the ECM is a loose, open network structure that can facilitate cell proliferation, migration, and differentiation as well as promote tissue growth and development. During tissue injury repair, a loose, open ECM is reconstructed outside cells, facilitating the migration of cells to lesions and promoting cell/tissue repair [21]. The ECM scaffold is the “soil” and “signal” of tissue regeneration; it can separate cells from the surrounding matrix, and it fulfills a role as a barrier for material transport [22].

Thus, an ECM scaffold, a precise and ordered network structure, has unique tissue regeneration and repair advantages. It can effectively support tissue regeneration, guide tissue reconstruction, and promote endometrial repair and proliferation while preventing IUA. Accordingly, its application prospects are worthy of further investigation.

## Data Availability

All data generated or analyzed during this study are included in this article. Further enquiries can be directed to the corresponding author.
